# Dr. Webster and His Spiritual Adviser

**Published:** 1850-08

**Authors:** 


*Dr. Webster and his Spiritual Adviser.*—At the meeting of the Governor and Council to consult on Dr. Webster's case, last week, several individuals appeared in his behalf, and another meeting for the same purpose, intended to be the last, was appointed for Thursday of this week. In the Rev. Dr. Putnam's plea in behalf of a commutation of the sentence of death, he alludes to the barbarous manner in which the remains of the late Dr. Parkman were disposed of. He seems to think there was no special exhibition of hard-heartedness in the case, but that medical men in general have no sort of feeling in such matters, and can cut or slash the dead body with perfect *sang froid.* With the utmost deference to the rectitude of Dr. Putnam's intentions in endeavoring to soften the public sentiment respecting the atrocity alluded to, we cannot allow the profession to have feelings and actions attributed to them which do not in fact exist. The intelligent portion of the community know full well the importance of the study of anatomy, and that for any other purpose than the benefit of science dissections of dead bodies would never be made. Upon that portion of the community we have no fear that the assertions of Dr. P. will have any injurious influence; but among those who are less informed, it may produce unpleasant feelings towards medical men, and in divers ways may tend to bring reproach upon the profession. It is perhaps unnecessary for us to state, as we feel warranted in doing, in unequivocal terms, that the statements alluded to misrepresent the character and feelings of the profession entirely. The whole fault in this melancholy case lies at the door of the one who now confesses that he perpetrated the tragedy. If this same confession had been made immediately after the homicide, no one can for a moment doubt that the result would have been entirely different from what it now is.

